# Crystal Orientation of Poly(l-Lactic Acid) Induced by Magnetic Alignment of a Nucleating Agent

**DOI:** 10.3390/polym10060653

**Published:** 2018-06-11

**Authors:** Ryosuke Kusumi, Sachi Teranishi, Fumiko Kimura, Masahisa Wada, Tsunehisa Kimura, Yoshiki Horikawa, Takahiko Kawai

**Affiliations:** 1 Division of Forest and Biomaterials Science, Kyoto University, Kyoto 606-8502, Japan; teranishi.sachi@gmail.com (S.T.); fkimura@kais.kyoto-u.ac.jp (F.K.); wadam@kais.kyoto-u.ac.jp (M.W.); kimura.tsunehisa.33e@st.kyoto-u.ac.jp (T.K.); 2 Department of Plant and Environmental New Resources, College of Life Sciences, Kyung Hee University, 1732 Deogyeong-daero, Giheung-gu, Yongin-si, Gyeonggi-do 446-701, Korea; 3 Institute of Agriculture, Tokyo University of Agriculture and Technology, Fuchu 183-8509, Japan; horikaw@cc.tuat.ac.jp; 4 Graduate School of Science and Technology, Gunma University, Ota 376-0053, Japan; kawaitakahiko@gunma-u.ac.jp

**Keywords:** poly(l-lactic acid), nucleating agent, magnetic orientation, epitaxial growth

## Abstract

The orientation of poly(l-lactic acid) (PLLA) crystals was controlled through crystal growth from a magnetically oriented nucleating agent, phenylphosphonic acid zinc (PPAZn). The one-dimensional magnetically oriented microcrystal array of PPAZn microcrystals revealed the relationship between the magnetization and crystallographic axes in the PPAZn crystal. The PPAZn microcrystals were homogeneously dispersed in PLLA via melt mixing, which decreased the molecular weight of the PLLA component due to degradation. The PPAZn microcrystals in the molten PLLA were uniaxially aligned under an 8-T static or rotating magnetic field. The wide-angle X-ray diffraction and small-angle X-ray scattering patterns of the PPAZn/PLLA composite films crystallized under each magnetic field showed that the PLLA lamellae grew from the surface of the PPAZn microcrystals, which were uniaxially oriented along the easy- or hard-magnetization axis, with the *c*-axis of PLLA parallel to the *bc*-plane of PPAZn. It was also suggested that the greater nucleating effect of PPAZn on PLLA was derived not from geometrical matching, but from factors such as favorable interactions and/or the plate-like shape of the microcrystal.

## 1. Introduction

Poly(l-lactic acid) (PLLA) is a biodegradable/biocompatible polyester that can be obtained from renewable resources. PLLA is also attractive as an advanced material, having chirality and piezoelectricity. For the optimization of these properties, precise and versatile control of the structure is crucial. Magnetic processing can be a powerful means of controlling the orientation of crystalline polymers [1]. It was demonstrated that crystalline polymers, including isotactic polystyrene [2,3] and poly(ethylene terephthalate) [4], undergo alignment when a high magnetic field is applied during melt crystallization [5,6]. It was also reported [7,8] that the alignment of polymer crystals was induced by epitaxial growth on a nucleating agent that was magnetically aligned in the polymer melt. The magnetic alignment of PLLA containing octamethylenedicarboxylic dibenzoylhydrazide (OMBH) [9] and phenylphosphonic acid zinc (PPAZn) [10] was achieved via melt crystallization under a static magnetic field. This indirect method using nucleating agents can be an alternative to the direct method, as some crystalline polymers insufficiently align themselves under magnetic fields, while nucleating agents easily undergo magnetic alignment.

In the presented study, we achieved control of the orientation of PLLA crystals through crystal growth on a magnetically oriented nucleating agent under static and rotating magnetic fields. PPAZn microcrystalline powder was chosen as a nucleating agent because PPAZn, which belongs to the monoclinic system, shows excellent nucleating effects on PLLA crystallization [11]. Biaxial crystals, such as the monoclinic system, have three different principal values (*χ*_1_ > *χ*_2_ > *χ*_3_) of the magnetic susceptibility tensor. Under a static magnetic field, the easy-magnetization axis (*χ*_1_) is uniaxially aligned parallel to the external magnetic field. On the other hand, the hard-magnetization axis (*χ*_3_) uniaxially aligns parallel to the rotation axis under a rotating magnetic field. Before the application of magnetic fields to the PPAZn/PLLA composite, one-dimensional magnetically oriented microcrystal arrays (1D-MOMAs) were prepared from the PPAZn microcrystalline powder, using static and rotating magnetic fields to investigate the relationship between the magnetization and crystallographic axes of a PPAZn crystal. Subsequently, the PPAZn/PLLA composite was subjected either to a static or a rotating magnetic field. Wide-angle X-ray diffraction (WAXD) and small-angle X-ray scattering (SAXS) measurements were carried out to evaluate the crystal orientation of PLLA.

## 2. Materials and Methods

PPAZn microcrystalline powder (Ecopromote) and UV-curable monomer (XVL-90K, viscosity 3.2 Pa·s) were kindly supplied by Nissan Chemical Industries Ltd. (Tokyo, Japan) and Kyoritsu Chemical and Co. Ltd. (Tokyo, Japan), respectively. PLLA (Biofront HL-L20) was purchased from Teijin Ltd. (Tokyo, Japan). The basic data of the average molecular weights (*M*_w_ and *M*_n_) and thermal properties (glass-transition (*T*_g_) and melting (*T*_m_) temperatures) are summarized in Table 1, together with those of the PLLA component in the PPAZn/PLLA composite (PPAZn concentration = 1%). 

Two types of 1D-MOMAs were prepared from the PPAZn microcrystalline powder: one involved the use of static magnetic fields, and the other, rotating magnetic fields. The PPAZn microcrystalline powder was pulverized with a mortar for 20 min, and dispersed into the UV-curable monomer. The weight fraction of PPAZn was approximately 10 wt %. The PPAZn/monomer mixture was allowed to stand for five days, in an effort to precipitate the larger crystals and aggregates. A small quantity of the suspension was taken from the upper layer, and poured into a plastic tube. The tube was mounted on a sample-rotating unit inside a cryogen-free superconducting magnet (Sumitomo Heavy Industries, Ltd., Tokyo, Japan), generating an 8-T horizontal static magnetic field. The tube was then rotated at 40 rpm about a vertical axis. The rotation of a sample under a static magnetic field is regarded to be equivalent to applying a rotating magnetic field to a stationary sample. For the preparation of the sample under a static magnetic field, the tube was placed on the same rotating unit, but rotation was not applied. After being exposed to the magnetic field for 50 min, the suspension was irradiated with UV light for 20 min to photopolymerize the UV-curable monomer. The consolidated specimen was removed from the tube to obtain the 1D-MOMA. The 1D-MOMAs prepared under static and rotating magnetic fields are coded as 1Ds-MOMA and 1Dr-MOMA, respectively.

PPAZn/PLLA composite films with different weight ratios were prepared according to the procedure described below. The PPAZn microcrystalline powder was dispersed in chloroform by vigorous stirring. The PLLA was then dissolved in the solution with 24 h of stirring. The weight ratios of PPAZn to PLLA were 0.01 and 0.1. The solvent was evaporated at room temperature for one week, followed by drying at 40 °C in vacuo for 12 h. The mixture obtained in bulk form was kneaded using a twin rotary mixer (Labo Plastmill 4C150, Toyo Seiki Seisaku-sho, Ltd., Tokyo, Japan) at 100 rpm and 180 °C for 15 min. The melt-mixed composite was hot-pressed at 200 °C, and then transferred to another compressing apparatus, before being cold-pressed at 20 °C so as to obtain a film of approximately 0.1-mm thickness. Square specimens (10 × 10 mm^2^) were cut off from the molded film, and subjected to magnetic treatment. The molecular weights and thermal properties of the PLLA component in the PPAZn/PLLA composite (PPAZn concentration = 1%) are shown in Table 1. The molecular weight of the PLLA component decreased considerably, due to degradation during the melt mixing. Therefore, we should note that the mechanical properties were changed from the original PLLA.

The PLLA and PPAZn/PLLA composite films were exposed to static and rotating magnetic fields (i.e., the sample was rotated in a static magnetic field). In the experiment under a static magnetic field, a specimen was kept in a home-built furnace placed in the bore center of a superconducting magnet (the same magnet as previously described), generating an 8-T static horizontal magnetic field. The direction of the magnetic field was parallel to the film surface. The specimen was heated to 200 °C under the 8-T static magnetic field for 10 min, followed by gradual cooling to room temperature at a rate of approximately −6 °C/min. It was confirmed via differential scanning calorimetry (without magnetic field) that the degree of crystallinity was ca. 40% for the PPAZn/PLLA film (PPAZn concentration = 1%), compared to 6% for neat PLLA heat-treated under the same conditions. The higher crystallinity of the PPAZn/PLLA film was caused by not only adding the nucleating agent, but also the decrease in molecular weight after melt mixing (see Table 1).

The obtained PLLA and PPAZn/PLLA films are denoted by PLLA-8TS and PPAZn/PLLA-8TS, respectively. A control experiment was also conducted for the PPAZn/PLLA specimen without the magnetic field (hereafter abbreviated as PPAZn/PLLA-0T).

In the case of the application of a rotating magnetic field, a specimen was set on a sample-rotating unit equipped with a furnace, and rotated at 40 rpm with the sample-rotation axis perpendicular to the magnetic field. The heating and cooling processes were the same as those followed for the static magnetic field. The PPAZn/PLLA film exposed to a rotating magnetic field is hereafter abbreviated as PPAZn/PLLA-8TR.

The morphology of the PPAZn microcrystals was observed under a transmission electron microscope (TEM). A JEOL JEM-2000EXII (Tokyo, Japan) was used at an acceleration voltage of 100 kV. Diffraction patterns were also recorded from an area 2 to 3 μm in diameter.

The observations of the PLLA crystalline phase in the PLLA and PPAZn/PLLA films were carried out using a Nikon OPTIPHOT-2 POL (Tokyo, Japan) polarizing optical microscope. A film specimen put on a slide glass was observed under crossed polars, with a 530-nm-sensitive tint plate. 

The WAXD measurements were conducted in reflection mode to investigate the crystal modification of PLLA in the composite films after exposure to the magnetic fields. A Rigaku Ultima IV with nickel-filtered Cu Kα radiation was used. The 2θ-scans were performed at ambient temperature (20 °C) from 5° to 25° with a step size of 0.02°. 

The crystal orientations of the PPAZn microcrystals in 1D-MOMAs, and PLLA in the magnetically oriented PPAZn/PLLA films were evaluated using WAXD measurements in transmission mode. The two-dimensional (2D) WAXD images were collected on a Rigaku RAXIS RAPID II system (Tokyo, Japan) equipped with an imaging plate (IP) using graphite-monochromatized Cu Kα radiation. The camera length between the sample and the IP was 127 mm. The ω scans were performed at ambient temperature (20 °C) from −15° to 15°.

The SAXS measurements were performed using the synchrotron X-ray source at BL40B2 in SPring-8 (Japan Synchrotron Radiation Research Institute, Hyogo, Japan). The X-ray beam was monochromatized to a wavelength *λ* = 1.2 Å. The beam center, detector tilt, and sample-to-detector distance (*L* = 1741.4 mm) were calibrated using a silver behenate standard. The SAXS patterns were recorded on an ORCA-Flash4.0 V2 Digital CMOS camera (Hamamatsu Photonics K.K., Hamamatsu, Japan).

## 3. Results and Discussion

### 3.1. Transmission Electron Microscope (TEM) Observation of Phenylphosphonic Acid Zinc (PPAZn) Microcrystals

**Figure 1:**
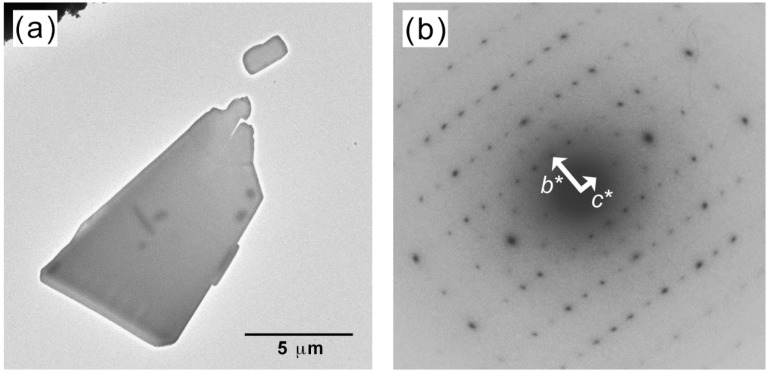
(**a**) Electron micrograph of a phenylphosphonic acid zinc (PPAZn) crystal, and (**b**) the corresponding diffraction pattern.

The shape of the PPAZn microcrystal, which is an important factor for the crystal growth of PLLA on the surface, was observed under TEM. The typical image of a PPAZn crystal is shown in Figure 1, along with the corresponding electron diffraction pattern. As can be seen in Figure 1a, PPAZn is a plate-like crystal with a size of 10 μm or less, and a thickness of approximately 50 nm. On the diffraction pattern in Figure 1b, two sets of layer lines were clearly observed in directions orthogonal to each other. From the crystallographic data of PPAZn (monoclinic, *a* = 14.47, *b* = 5.17, *c* = 10.56 Å, and β = 94.79°) [10], these diffraction spots were assigned to the [010] and [001] directions. Therefore, it was concluded that the *a*-axis of PPAZn was almost perpendicular to the larger plane, whereas the *b*-axis (two-fold axis) was parallel to the plane. These results indicated that the *bc*-plane was located on the larger surface of the crystal.

### 3.2. Orientation of PPAZn Microcrystals under Static and Rotating Magnetic Fields

**Figure 2:**
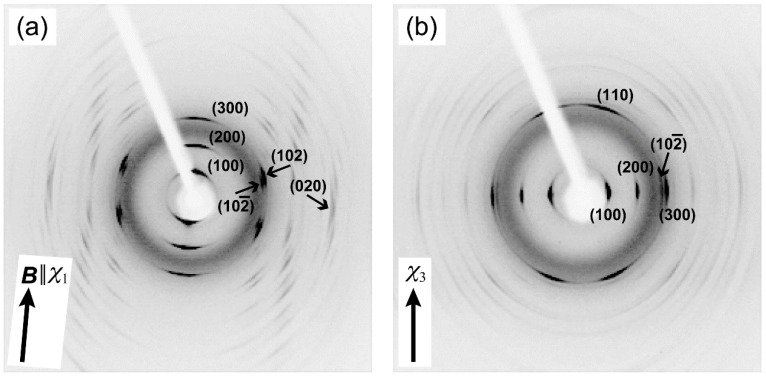
Two-dimensional wide-angle X-ray diffraction (2D-WAXD) patterns of one-dimensional magnetically oriented microcrystal arrays (1D-MOMAs) of PPAZn. (**a**) 1D-MOMA prepared under a static magnetic field (1Ds-MOMA), and (**b**) 1D-MOMA prepared under a rotating magnetic field (1Dr-MOMA). X-rays were impinged perpendicular to the *χ*_1_-axis and *χ*_3_-axis for (**a**) and (**b**), respectively.

**Figure 3:**
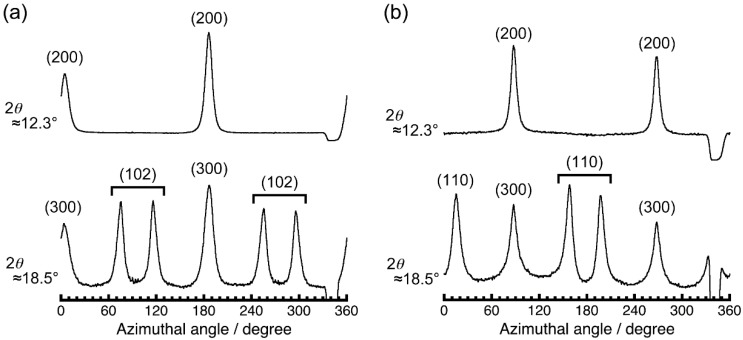
Azimuthal plots of (**a**) 1Ds-MOMA, and (**b**) 1Dr-MOMA. The β-scans were performed at 2θ ≈ 12.3° and 18.5° for each 2D pattern in Figure 2. β = 0° and 180° correspond to the vertical direction in the diffraction images.

Before magnetic alignment of PPAZn/PLLA composite films, PPAZn 1D-MOMAs were prepared under static and rotating magnetic fields in order to determine the relationship between the magnetic susceptibility and the crystallographic axes. Figure 2 shows the 2D-WAXD images obtained for 1D-MOMAs. Fiber diffraction patterns were clearly observed for both MOMAs. The assignment of each diffraction spot was based on the crystallographic data of PPAZn [10]. The azimuthal profiles scanned at 2θ ≈ 12.3° and 18.5° are shown in Figure 3. The half-width of the diffraction spots was approximately 9.6°.

**Figure 4:**
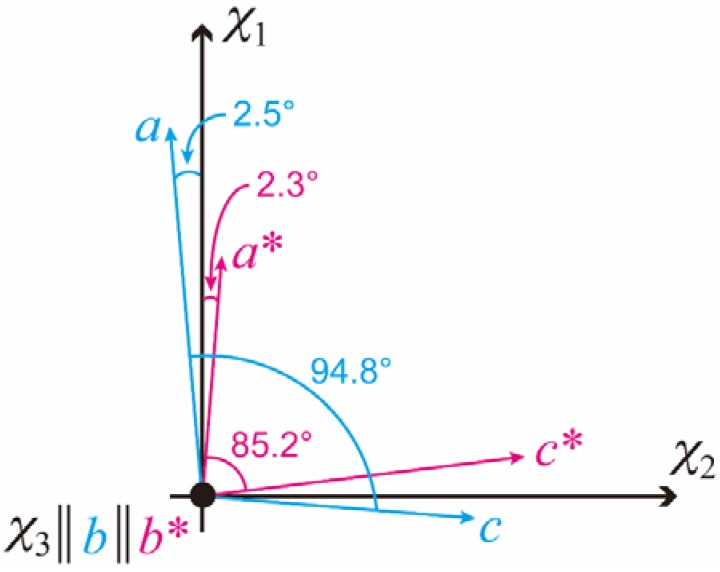
Schematic diagram showing the relationships between the magnetization and crystallographic axes of a PPAZn crystal in real and reciprocal spaces.

As can be seen in Figure 2a, 1Ds-MOMA exhibited (020) diffractions in the equatorial direction. For 1Dr-MOMA, the diffraction from the (020) plane was never detected when the X-rays were irradiated perpendicular to the *χ*_3_ axis (Figure 2b). Because the PPAZn crystal belongs to the monoclinic system, the *b*-axis (two-fold axis) should be one of the magnetic susceptibility axes [12]. Thus, we concluded that the *b*-axis coincided with the hard-magnetization axis (*χ*_3_). For both 1Ds-MOMA and 1Dr-MOMA, (100), (200), and (300) diffractions were detected. The 1Ds-MOMA exhibited these diffractions in the direction of the magnetic field, whereas these diffractions appeared in the equatorial direction for 1Dr-MOMA. Therefore, the a*-axis of PPAZn was expected to be close to the easy-magnetization axis (*χ*_1_). The direction of the a*-axis could be precisely determined using the four (102) diffractions in Figure 2a. From the comparison with the *χ*_1_ direction that was perpendicular to the (020) direction (‖ *χ*_3_ axis), the deviation angle between the two directions was estimated at 2.3°. Based on the above results, the crystallographic axes could be related with the magnetization axes. Figure 4 illustrates the relationships between the magnetization and the unit-cell axes in real and reciprocal spaces. The deviation of the *a*-axis from the *χ*_1_-axis was determined to be 2.5°.

### 3.3. Crystal Orientation of Poly(l-Lactic Acid) (PLLA) Induced by Magnetically Aligned PPAZn

**Figure 5:**
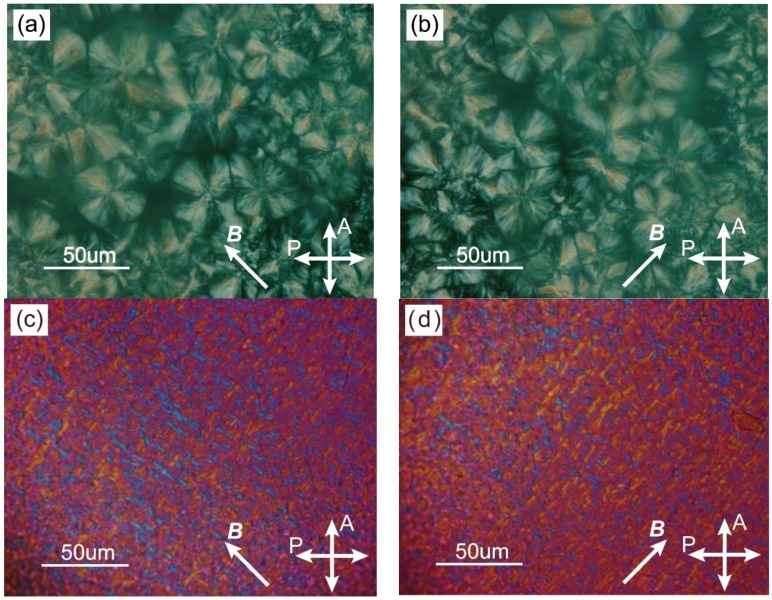

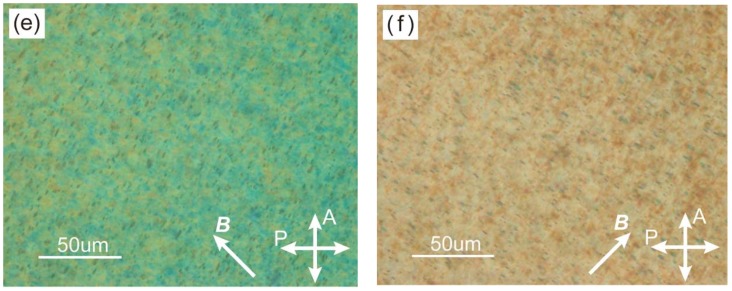
Polarized optical micrographs of (**a**,**b**) poly(l-lactic acid) under the 8-T static magnetic field (PLLA-8TS); (**c**,**d**) PPAZn/PLLA under no magnetic field (PPAZn/PLLA-0T); and (**e**,**f**) PPAZn/PLLA under the 8-T static magnetic field (PPAZn/PLLA-8TS). The micrographs of each sample were taken under the two optical settings indicated in each figure.

Figure 5 shows the polarized optical micrographs of the PLLA-8TS, PPAZn/PLLA-0T, and PPAZn/PLLA-8TS specimens. As shown in Figure 5a,b, spherulites with Maltese-cross patterns were observed for PLLA-8TS. This result indicated that PLLA only could not undergo magnetic alignment. For the PPAZn/PLLA-0T containing a nucleating agent that was not exposed to the magnetic field, there was no change in color upon rotation of the film under crossed polars, as shown in Figure 5c,d. On the other hand, for the PPAZn/PLLA-8TS containing a nucleating agent that was exposed to the magnetic field, color changes were observed, as shown in Figure 5e,f, depending on the angle between the direction of the applied magnetic field and the positions of the crossed polars, indicating that PLLA was aligned.

**Figure 6:**
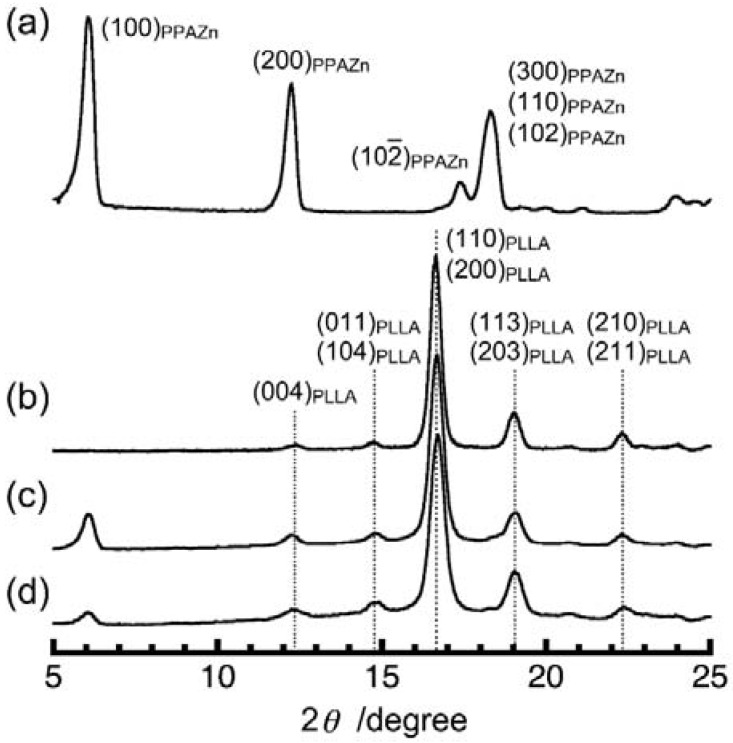
Powder X-ray diffraction (XRD) patterns of (**a**) PPAZn microcrystalline powder, (**b**) neat PLLA which was melt crystallized at 140 °C for 2 h, (**c**) PPAZn/PLLA-0T, and (**d**) PPAZn/PLLA-8TS.

It was reported that PLLA crystals take four different crystal modifications (α, β, γ, and δ (α′)) depending on crystallization conditions [13,14,15,16,17,18,19,20]. The effects of the surface of PPAZn, and the presence of the magnetic field on the crystal modification of PLLA crystals were investigated. Figure 6 shows the powder X-ray diffraction (XRD) patterns obtained for PPAZn microcrystalline powder, neat PLLA, PPAZn/PLLA-0TS, and PPAZn/PLLA-8TS. The measurement of neat PLLA was carried out following isothermal melt crystallization at 140 °C for 2 h. Under this condition, PLLA formed the most common α phase that had two distorted 10/3 helical chains in the pseudo-orthogonal unit cell (*P*2_1_2_1_2_1_, *a* = 10.683, *b* = 6.170, and *c* = 28.860 Å) [15]. Figure 6 shows that both PPAZn/PLLA-0T and PPAZn/PLLA-8TS exhibited the same α-form pattern as neat PLLA. This clearly showed that the presence of PPAZn and magnetic fields did not affect the formation of the *α* phase.

**Figure 7:**
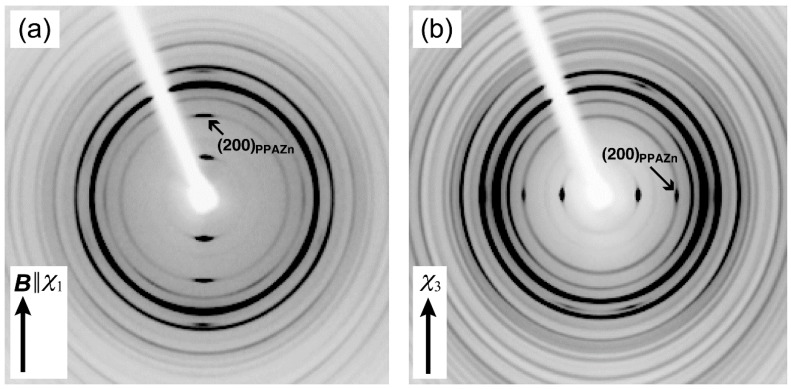
2D-WAXD patterns of (**a**) PPAZn/PLLA-8TS, and (**b**) PPAZn/PLLA under the 8-T rotating magnetic field (PPAZn/PLLA-8TR). The X-rays were impinged perpendicular to the *χ*_1_-axis and *χ*_3_-axis for (**a**) and (**b**), respectively, of the PPAZn crystal.

**Figure 8:**
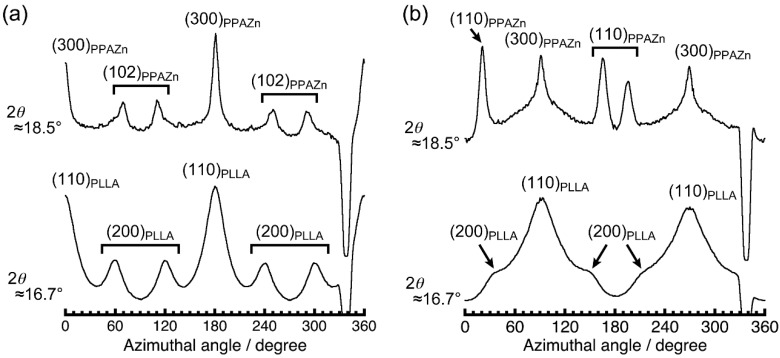
Azimuthal β-scans of (**a**) PPAZn/PLLA-8TS with the 0–180° direction parallel to the direction of the magnetic field (‖ *χ*_1_ direction of PPAZn), and (**b**) PPAZn/PLLA-8TR with the 0–180° direction parallel to the direction of the sample rotation axis (‖ *χ*_3_ direction of PPAZn). The β-scans were performed at 2θ ≈ 18.5° and 16.7° for each 2D pattern in Figure 7.

To evaluate the crystal orientation of PLLA, and its correlation with the magnetic alignment of PPAZn microcrystals, WAXD measurements were performed in transmission mode. Figure 7 shows the 2D-WAXD images obtained for PPAZn/PLLA-8TS and PPAZn/PLLA-8TR films. The azimuthal plots scanned at 2θ ≈ 18.5° and 16.7° are also shown in Figure 8. The diffraction spots from PPAZn (e.g., (200) diffraction) were clearly detected for both films, indicating that the PPAZn microcrystals were magnetically aligned in the molten PLLA. The half-widths of the spots, which were determined from the diffraction peaks in Figure 8, were approximately 11.2° and 10.1° for the PPAZn/PLLA-8TS and PPAZn/PLLA-8TR films, respectively. The diffraction patterns were identical to those of 1Ds-MOMA and 1Dr-MOMA in Figure 2 and Figure 3, respectively, indicating that PPAZn crystals were uniaxially aligned in the PPAZn/PLLA films, with the *a*-axis almost parallel to the *χ*_1_-axis under the static magnetic field, whereas the *b*-axis aligned parallel to the *χ*_3_-axis under the rotating magnetic field. This indicated that the alignment manner of PPAZn was not altered during crystallization of the PPAZn/PLLA mixture.

In contrast to the sharp diffraction spots from PPAZn, the diffraction patterns from the PLLA samples appeared as rings on the 2D images. However, their azimuthal plots clearly exhibited the intensity distributions, as shown in Figure 8. For the PPAZn/PLLA-8TS film, the diffraction from the (110)_PLLA_ plane appeared at 0° and 180°, that is, in the direction of the static magnetic field (*χ*_1_ direction). For the PPAZn/PLLA-8TR film, the (110)_PLLA_ diffractions were detected in the direction perpendicular to the *χ*_3_-axis. For both films, the peak positions of (110)_PLLA_ diffraction coincided with those of (300)_PPAZn_. These results indicated that the (110)_PLLA_ plane was located perpendicular to the *a*_PPAZn_-axis (≈*χ*_1_-axis), and parallel to the *b*_PPAZn_-axis (=*χ*_3_-axis). In the crystallization process of PLLA, the folding of PLLA chains occurs along the direction of the reciprocal lattice vector, **G**(110)_PLLA_ [21]. Thus, the crystal growth of PLLA probably occurred from the *bc*-plane of the PPAZn crystal, with the (110)_PLLA_ plane parallel to it. This interpretation is reasonable because the *bc*-plane of PPAZn was almost parallel to the larger surface of the crystal, as discussed in Section 3.1.

**Figure 9:**
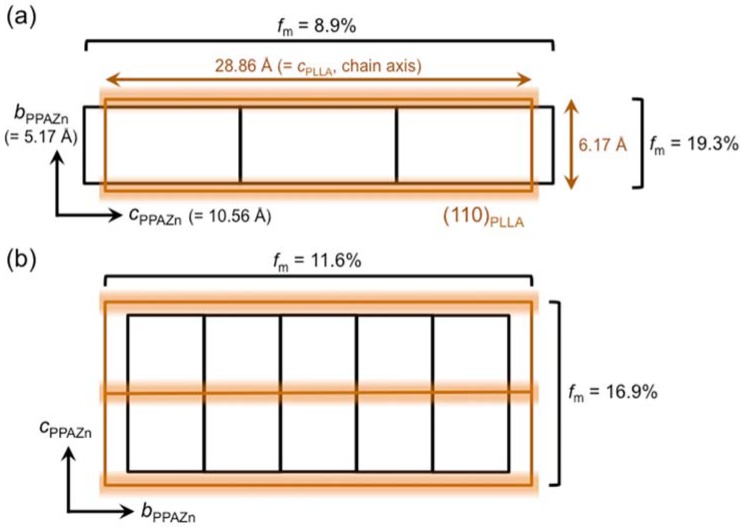
Lattice matching between the (110)_PLLA_-plane and the *bc*_PPAZn_-plane under the assumptions of (**a**) *c*_PPAZn_ being parallel to *c*_PLLA_ (*c*_PPAZn_‖*c*_PLLA_) and (**b**) *b*_PPAZn_‖*c*_PLLA_.

The lattice matching between the (110)_PLLA_-plane and the *bc*_PPAZn_-plane was evaluated in order to clarify the direction of the *c*-axis (chain axis) of PLLA on the *bc*_PPAZn_-plane. The misfit factor (*f*_m_), which provides the degree of conformity at the contact plane, was estimated by the following equation:

*f*_m_ = (*L*_d_ − *L*_s_)/*L*_s_ × 100, (1)

where *L*_s_ and *L*_d_ are the lattice parameters of a substrate and deposit, respectively. As can be seen from the *f*_m_ values in Figure 9, there was almost no difference in the matching between the two possible arrangements. Furthermore, evaluation from the view point of geometry requires matching at the molecular level; however, the atomic coordinates of a unit cell of PPAZn were not determined. Considering the positions of diffractions from PLLA in the 2D-WAXD patterns (Figure 8), it may be assumed that the *c*-axis of PLLA was located parallel to the *c*-axis of PPAZn. However, we could not prove that conclusion based only on the WAXD patterns, as a random orientation of the PLLA *c*-axis on the PPAZn *bc*-plane also provided the same diffraction patterns, as shown in Figure 8. Therefore, we measured the SAXS images for the magnetically aligned PPAZn/PLLA films.

**Figure 10:**
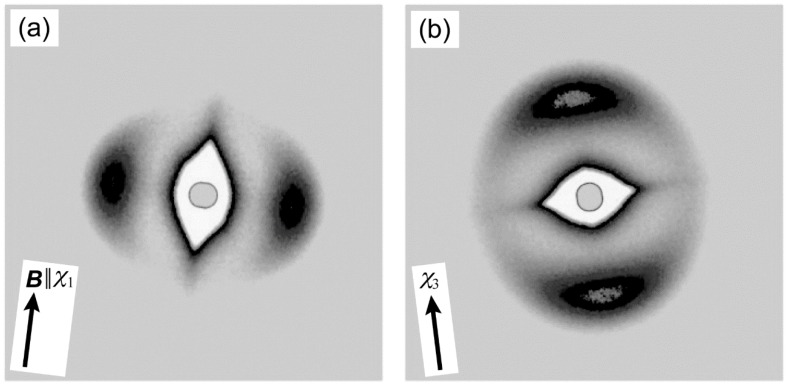
Small-angle X-ray scattering (SAXS) images of (**a**) PPAZn/PLLA-8TS, and (**b**) PPAZn/PLLA-8TR. The directions of the impinging X-rays were perpendicular to the *χ*_1_-axis and *χ*_3_-axis for (**a**) and (**b**), respectively.

Figure 10 shows the SAXS images obtained for the PPAZn/PLLA-8TS and PPAZn/PLLA-8TR films. For PPAZn/PLLA-8TS, the scatterings from the stacked lamellae of PLLA appeared perpendicular to the *χ*_1_-axis. On the other hand, the PPAZn/PLLA-8TR film exhibited scattering spots in the direction parallel to the *χ*_3_-axis. The above assumption that the *c*-axis of PLLA was located parallel to the *c*-axis (≈*χ*_2_-axis) of PPAZn was negated, because the scatterings of PPAZn/PLLA-8TR would be detected in the direction perpendicular to the *χ*_3_-axis. The latter result seemed to suggest that the PLLA lamellae were stacked in the *χ*_3_ direction (i.e., the *c*-axis of PLLA lay parallel to the *b*-axis of PPAZn); however, this model conflicted with the WAXD pattern of PPAZn/PLLA-8TR. In this model, (200)_PLLA_ diffraction peaks would appear at the same positions as the (110)_PLLA_ peaks in the azimuthal plot; however, the (200)_PLLA_ peaks were actually detected at positions shifted by 60° from the (110)_PLLA_ peaks (Figure 8b). Thus, the *c*-axis of PLLA was neither located parallel to the *c*-axis nor to the *b*-axis of PPAZn, but instead, randomly distributed on the *bc*-plane of PPAZn. 

The epitaxy between a deposit and the substrate is mainly classified into the following three types: “graphoepitaxy”, “soft epitaxy”, and “hard epitaxy” [22]. For soft and hard epitaxies, the mismatch at a contact plane must not exceed 15% and 10%, respectively. Therefore, larger *f*_m_ values (>15%) shown in Figure 9 potentially indicated a random distribution (“graphoepitaxy”).

**Figure 11:**
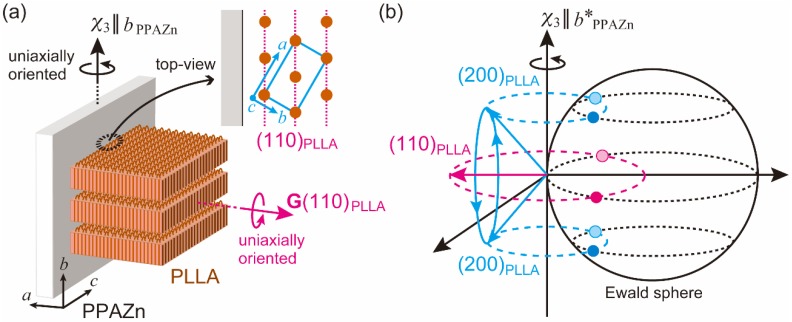
Schematic diagrams explaining the diffraction patterns obtained for PPAZn/PLLA-8TR. (**a**) The PLLA lamellae grew from the surface of PPAZn with the *c*-axis (chain axis) of PLLA parallel to the *bc*-plane of PPAZn. The stacked lamellae were uniaxially oriented along the direction of the reciprocal vector, **G**(110)_PLLA_. In addition, the PPAZn substrate uniaxially aligned along the χ_3_-axis (‖ *b*-axis) under a rotating magnetic field. (**b**) Under this condition, the **G**(200)_PLLA_ vectors were randomly distributed along the **G**(110)_PLLA_ oriented uniaxially along the *χ*_3_-axis (‖ b*-axis) of the substrate, resulting in two diffraction spots of (110)_PLLA_ on the equator, and four diffraction spots of (200)_PLLA_ at positions shifted by approximately 60° from the (110)_PLLA_ diffraction spots.

Figure 11a illustrates the crystal growth of PLLA from the magnetically aligned PPAZn under the rotating magnetic field. The *c*-axes (chain axes) of PLLA were uniformly distributed on the *bc*-plane of PPAZn, with the (110)_PLLA_ plane parallel to the surface of PPAZn. This condition implied a planar alignment of the *c*-axes of PLLA along **G**(110)_PLLA_. The *b*-axes (*χ*_3_-axes) of PPAZn were uniaxially aligned under the rotating magnetic field, resulting in the four diffraction spots shown in Figure 11b. This predicted pattern coincided with the WAXD image in Figure 8b. Simultaneously, this model produced a planar alignment of the lamellar stacks along the direction of the lamellar growth (‖ **G**(110)_PLLA_). With the uniaxial alignment of the PPAZn substrate along the *b*-axis, the scatterings from the stacked PLLA lamellae were distributed in all directions, but the intensity of the scattering became higher at the poles, as can be seen in Figure 10b. This orientation model was also compatible with the WAXD and SAXS patterns obtained for the PPAZn/PLLA-8TS film. The unique orientation observed in the PPAZn/PLLA system is similar to the magnetic alignment of cellulose whiskers containing nanocrystals oriented uniaxially about the long axis of the whisker [23].

**Figure 12:**
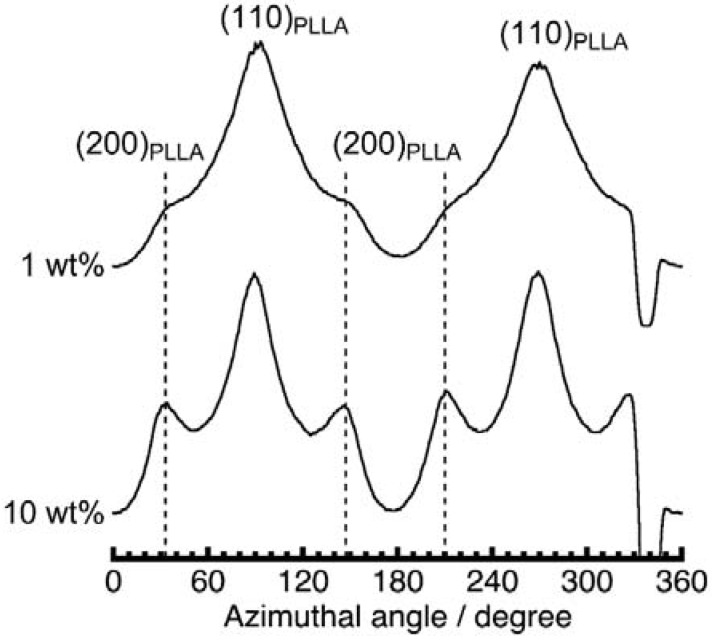
Comparison of the azimuthal plots scanned at 2θ ≈ 16.7° in the 2D-WAXD images of PPAZn/PLLA-8TR with both concentrations of PPAZn (1 and 10 wt %). The (200) diffraction peaks of PPAZn/PLLA-8TR containing 10 wt % PPAZn were more clearly detected than those containing 1 wt % PPAZn.

We provide further insight below into the reason for the uniform distribution of the PLLA lamellae on PPAZn, along the direction of lamellar growth. Considering the shape and unit-cell parameters of the PPAZn crystal, the following two processes were proposed: (A) twisting of the PLLA lamellae after crystallization on the *bc*-plane of PPAZn, according to the “hard epitaxial” manner, and (B) lamellar growth from the larger surface of PPAZn without such a “hard epitaxy” at the contact plane. In order to verify which of these was true, we performed a WAXD measurement for PPAZn/PLLA-8TR containing 10 wt % PPAZn. At such a high concentration of PPAZn, the growth of PLLA lamellae was limited to being in the vicinity of the nucleating agent. Figure 12 shows the azimuthal plot obtained at 2θ ≈ 16.7° for PPAZn/PLLA-8TR films with both PPAZn concentrations. If model (A) was true, the (200)_PLLA_ diffractions of the film of higher PPAZn concentrations would be absent, or detected only along the equator. Contrarily, in Figure 12, these peaks appeared more clearly at the same positions, indicating that model (A) was false. In the presented PPAZn/PLLA system, the crystallization of PLLA seemed to occur according to a “soft epitaxy”, which originated from some favorable interactions, rather than a “hard epitaxy”, which required geometrical matching at a molecular level. The higher nucleating effect of PPAZn may be ascribed to the combination of a “soft epitaxy” and the plate-like shape of the crystal.

Further discussion on epitaxy is still needed regarding the molecular packing of both compounds; however, the crystal structure of PPAZn is yet to be determined completely due to the difficulty of preparing a single crystal large enough for structural analysis. The magnetically oriented microcrystal array and suspension [24,25,26], which convert a microcrystalline powder into a “single crystal”-like composite, are suitable for the single-crystal diffraction analysis of such a compound. The epitaxy in the PPAZn/PLLA system can be discussed in detail following the precise determination of the packing of PPAZn molecules in a unit cell.

## 4. Conclusions

In the presented study, we performed uniaxial alignment of PLLA crystals via crystal growth on magnetically oriented PPAZn under static and rotating magnetic fields. The PPAZn microcrystals were homogeneously dispersed in PLLA through melt mixing, which caused a decrease in the molecular weight of the PLLA component in the composite. It was successfully demonstrated that the orientation of PLLA could be controlled via the magnetic alignment of the nucleating agent. The orientation control presented here may be achieved using other nucleating agents such as zinc phenylphosphonate monohydrate [27] and OMBH [28]. It was also revealed that the higher nucleating effect of PPAZn on PLLA is ascribed to the combination of some favorable interactions, and the plate-like shape of the nucleating agent, rather than geometrical matching at a molecular level.

## Figures and Tables

**Table 1:** Molecular weights and thermal transition data of neat poly(l-lactic acid) (PLLA) and the PLLA component in the phenylphosphonic acid zinc (PPAZn)/PLLA composite.

Sample	*M*_w_^(a)^	*M*_n_^(a)^	*M*_w_/*M*_n_^(a)^	*T*_g_^(b)^/°C	*T*_m_^(b)^/°C
neat PLLA	147,000	40,000	3.68	61.2	176.2
melt-mixed PLLA	77,000	17,000	4.53	62.7	174.9

^(a)^ Determined using gel permeation chromatography (mobile phase, 0.25 mL/min tetrahydrofuran at 40 °C) with polystyrene standards; ^(b)^ Determined via differential scanning calorimetry in a nitrogen atmosphere. The glass-transition temperature (*T*_g_) and melting temperature (*T*_m_) were determined from the midpoint of a discontinuity and the peak-top position of a melting endotherm in heat flow, respectively, in the thermogram obtained in the second heating scan.

## References

[1] Kimura T. (2003). Study on the effect of magnetic fields on polymeric materials and its application. Polym. J..

[2] Ezure H., Kimura T., Ogawa S., Ito E. (1997). Magnetic orientation of isotactic polystyrene. Macromolecules.

[3] Ebert F., Thurn-Albrecht T. (2003). Controlling the orientation of semicrystalline polymers by crystallization in magnetic fields. Macromolecules.

[4] Kimura T., Kawai T., Sakamoto Y. (2000). Magnetic orientation of poly(ethylene terephthalate). Polymer.

[5] Naga N., Ishikawa G., Noguchi K., Takahashi K., Watanabe K., Yamato M. (2013). Magnetic-field induced alignment of low molecular weight polyethylene. Polymer.

[6] Suzuki K., Yamato M., Hirota N. (2014). Alignment of nylon 6 by melt crystallization in a high magnetic field. Kobunshi Ronbunshu.

[7] Kawai T., Iijima R., Yamamoto Y., Kimura T. (2001). Crystal orientation of *N*,*N*′-Dicyclohexyl-2, 6-naphthalenedicarboxamide in High Magnetic Field. J. Phys. Chem. B.

[8] Kawai T., Iijima R., Yamamoto Y., Kimura T. (2002). Crystal orientation of β-phase isotactic polypropylene induced by magnetic orientation of *N*,*N*′-dicyclohexyl-2, 6-naphthalenedicarboxamide. Polymer.

[9] Yamato M., Kudo Y., Takahashi K., Watanabe K., Kawamoto N. (2011). Magnetic alignment of poly(l-lactic acid) containing a nucleating agent. Chem. Lett..

[10] Inamura M., Okano R., Kishi T., Awano H., Takahashi T., Yonetake K. Epitaxy between polymer and nuclear agent under magnetic field and analysis of the fine structure. Proceedings of the 5th Annual Meeting of the Magneto-Science Society of Japan.

[11] Suryanegara L., Okumura H., Nakagaito A.N., Yano H. (2011). The synergetic effect of phenylphosphonic acid zinc and microfibrillated cellulose on the injection molding cycle time of PLA composites. Cellulose.

[12] Nye J.F., Nye J.F. (1985). The groundwork of crystal physics. Physical Properties of Crystals: Their Representation by Tensors and Matrices.

[13] De Santis P., Kovacs A.J. (1968). Molecular conformation of poly(s-lactic acid). Biopolymers.

[14] Sasaki S., Asakura T. (2003). Helix distortion and crystal structure of the α-form of poly(l-lactide). Macromolecules.

[15] Wasanasuk K., Tashiro K., Hanesaka M., Ohhara T., Kurihara K., Kuroki R., Tamada T., Ozeki T., Kanamoto T. (2011). Crystal structure analysis of poly(l-lactic acid) α form on the basis of the 2-dimensional wide-angle synchrotron X-ray and neutron diffraction measurements. Macromolecules.

[16] Hoogsteen W., Postema A.R., Pennings A.J., ten Brinke G., Zugenmaier P. (1990). Crystal structure, conformation, and morphology of solution-spun poly(l-lactide) fibers. Macromolecules.

[17] Puiggali J., Ikada Y., Tsuji H., Cartier L., Okihara T., Lotz B. (2000). The frustrated structure of poly(l-lactide). Polymer.

[18] Cartier L., Okihara T., Ikada Y., Tsuji H., Puiggali J., Lotz B. (2000). Epitaxial crystallization and crystalline polymorphism of polylactides. Polymer.

[19] Zhang J., Tashiro K., Domb A.J., Tsuji H. (2006). Confirmation of disorder α form of poly(l-lactic acid) by the X-ray fiber pattern and polarized ir/raman spectra measured for uniaxially-oriented samples. Macromol. Symp..

[20] Wasanasuk K., Tashiro K. (2011). Crystal structure and disorder in poly(l-lactic acid) δ form (α′ form) and the phase transition mechanism to the ordered α form. Polymer.

[21] Miyata T., Masuko T. (1997). Morphology of poly(l-lactide) solution-grown crystals. Polymer.

[22] Thierry A., Lotz B.A., Piorkowska E., Rutledge G.C. (2013). Epitaxial crystallization of polymers: Means and issues. Handbook of Polymer Crystallization.

[23] Song G., Kimura F., Kimura T., Piao G. (2013). Orientational distribution of cellulose nanocrystals in a cellulose whisker as studied by diamagnetic anisotropy. Macromolecules.

[24] Kimura T., Tamura R., Miyata M. (2015). Magnetically oriented microcrystal arrays and suspensions: Application to diffraction methods and solid-state NMR spectroscopy. Advances in Organic Crystal Chemistry.

[25] Tsukui S., Kimura F., Garman E.F., Baba S., Mizuno N., Mikami B., Kimura T. (2016). X-ray crystal structure analysis of magnetically oriented microcrystals of lysozyme at 1.8 Å resolution. J. Appl. Cryst..

[26] Tsuboi C., Aburaya K., Kimura F., Maeyama M., Kimura T. (2016). Single-crystal structure determination from microcrystalline powders (~5 μm) by an orientation attachment mountable on an in-house X-ray diffractometer. CrystEngComm.

[27] Pan P., Liang Z., Cao A., Inoue Y. (2009). Layered metal phosphonate reinforced poly(l-lactide) composites with a highly enhanced crystallization rate. ACS Appl. Mater. Interfaces.

[28] Kawamoto N., Sakai A., Horikoshi T., Urushihara T., Tobita E. (2007). Nucleating agent for poly(l-lactic acid)—An optimization of chemical structure of hydrazide compound for advanced nucleation ability. J. Appl. Polym. Sci..

